# 

**Published:** 1900-01-20

**Authors:** 


**DEATH.**

We regret to announce the death of Miss Bertha Snape (sister), of the Wakefield Union Infirmary, from typhoid fever. Miss Snape trained at the Birmingham Infirmary, and has since held the post of sister at the above infirmary. Her death is greatly lamented by a large circle of friends. (674)

### NOTICE TO CORRESPONDENTS.

All MS., Letters, Books for Review, and other matters intended for the Editor should be addressed The Editor, "The Hospital," The Hospital Building, 28 & 29, Southampton Street, Strand, W.C.

The Editor cannot undertake to return rejected MS., even when accompanied by stamped directed envelope.

**Notice to Advertisers and Subscribers.**

All Advertisements, Orders for Copies of The Hospital, and Business Communications should be addressed to **THE MANAGER (Not to the Editor),** The Hospital, The Hospital Building, 28 & 29, Southampton Street, Strand, London, W.C.

The Cost of Subscription, post free, is as follows:—Per week, 2½d.; per quarter, 2s. 8d.; per half-year, 5s. 3d.; per annum, 10s. 6d. Foreign:—Per annum, 15s. 2d., payable, in all cases, in advance.

**NOTICE.—Vol. XXVI. of "The Hospital" (April, 1899, to September, 1899), in handsome cloth binding, is now on sale at the Office of this Journal, price 7s. 6d. Cases for binding the Numbers contained in this Volume 1s. 6d. Index to Vol. XXVI., 2½d., post free.**

**The Monthly Part for February will be ready on 1st February. Price 6d., by post 8d.**

### BOOKS RECEIVED.

T. Fisher Unwin.

"Unwin's Chap Book," 1899-1900.

Baillière, Tindall, and Cox.

"Aids to Materia Medica." Part III. By William Murrell, M.D., F.R.C.P.

Scientific Press.

"Mental Nursing." The Burdett Series, No. 9. By William Harding, M.D., M.R.C.P.

"Home Nursing of Sick Children." By J. D. E. Mortimer, M.D., F.R.C.S.

H. K. Lewis.

"Letter, Word, and Mind Blindness." By James Hinshelwood, M.A., M.D., F.F.P.S.

**Periodicals and Pamphlets.**—Middlesex Hospital, Medical Press, Sanitary Record, Journal of Education, Literary Digest Woman, The Zenana.

### SCRAPS AND GLEANINGS.

There is still a debt of £360 on the new buildings of the Royal Victoria Hospital, Bournemouth, which were recently opened.

During the past year 74 patients have been treated in the Carlisle Fever Hospital. An addition is to be made to the existing buildings consisting of two blocks containing accommodation for 12 beds. The cost, it is estimated, will be £1,800.

The Portland Ambulance, which left for the Cape on December 9th, carries a large supply of Bovril, the gift of a retired naval officer. The same officer has also generously supplied the hospital ships "Princess of Wales" and "Maine" with large quantities of Bovril.

At the beginning of this year the funds in the hands of the authorities of the Victoria Cottage Hospital, Guernsey, were only £8 11s. 5d. An appeal for increased support is being urgently made, as the hospital has been considerably enlarged since it was first started.

Liebig Company's Extract will henceforward bear a new name, "Lemco," which will appear on the wrappers and on the jar itself, in addition to the signature of Baron Justus von Liebig. This alteration of name will prevent any confusion between the extract prepared by the Liebig Company, who were the pioneers in the manufacture of extract, and others.

It is proposed to have 240 beds for scarlet fever patients, 60 beds for diphtheria patients, 88 beds for typhoid, and 48 beds for isolating purposes at the new Leeds Hospital buildings. This gives a total of 436 beds, for which a staff of 221 will be required. It has been resolved that application be made to the Local Government Board to borrow a sum of £300,000 for hospital purposes, in place of the loan of £200,000 which was applied for in September, and which has been found to be insufficient.

The Free Hospital for Sick Children, Nottingham, has had a handsome gift in the shape of a house and some four or five acres of land adjoining for the purpose of erecting an enlarged hospital on this site, which is stated to be one of the most suitable in the borough. In the 20 years between 1877 and 1897 the in-patients have increased four times in number, and the out-patients five times. For some time the authorities have contemplated enlargement, but have been prevented using the existing site owing to want of space and the locality being unsuitable, while they have not been able to obtain a fresh one. The gift in question is therefore most welcome and generous. The donor is Mr. T. J. Birkin.

### THE STUDENT OF MEDICINE.

### APPOINTMENTS.

N. C. Bailes, M.B., B.Ch.Durh., appointed Resident Surgeon to the General Dispensary, Birmingham. F. J. H. Bateman, B.A Cantab., M.B., C.M.Edin., appointed Resident Medical Officer to the Hertford British Hospital, Paris. J. Black, L.D.S Eng., appointed Dental House Surgeon to Guy's Hospital. H. T. Campkin, L.D.S.Eng., appointed Dental Surgeon to Guy's Hospital. S. G. Champion, M.B., C.M.Edin., appointed House Surgeon to the Grimsby and District Hospital. G. Cole, M.R.C.S., L.R.C.P., appointed Public Vaccinator for North-West District of Nottingham. S. R. Dyer, M.D.Brux., M.R.C.S.Eng., L.R.C.P.Lond., D.P.H.Eng., Barrister-at-Law of the Middle Temple, appointed Medical Officer to H.M. Prison, Stafford. J. S. McGowan, B.Sc Lond., M.D., appointed Medical Officer for the Fifth District of the Oldham Union. R. Morrison, L.R.C.P., L.R.C.S.Edin., appointed Medical Officer for the Duffield District of the Belper Union. J. J. Norton, L.R.C.S., L.K.Q.C.P.Irel., appointed Medical Officer for the Alrewas District of the Lichfield Union. J. Orton, jun., M.R.C.S., L.R.C.P.Lond., D.P.H.Edin., appointed Medical Officer for the Fourth District of the Coventry Union. F. J. Pearce, L.D.S.-Eng., appointed Dental Surgeon to Guy's Hospital. F. Rowland, L.R.C.P., L.R.C.S.Edin., appointed Resident Surgeon to the Birmingham General Dispensary. N. E. H. Scott, M.B., Ch.B.Glasg.Univ., appointed House Physician to the North-Eastern Hospital for Children, Hackney Road, Shoreditch, N.E. A. G. Young, M.B., appointed Medical Officer for the Castle Bytham District of the Bourne Union.

Newcastle Royal Infirmary.

The following appointments have been made: Senior House Physician, W. Simpson, M.B., B.S.Durh. Junior House Physician, J. Muirhead, M.B., B.S.Durh. House Surgeons, J. McConnell, M.B., B.S.Durh.; L. J. Blandford, M.B., B.S.Durh. Assistant House Surgeon, E. Gofton, M.B., B.S.Durh.

A NEW TEXT-BOOK FOR STUDENTS.

Crown 8vo., nearly 150 pp. Illustrated. Cloth. Price 2s. 6d.

**MEDICAL GYMNASTICS,** Including the SCHOTT (Nauheim) Movements. Being a Text-Book of Massage and Mechanical Therapeutics Generally, for Medical Students. By AXEL V. GRAFSTROM, B.Sc., M.D.

London: The Scientific Press, Ltd., 28 & 29, Southampton Street, Strand, W.C.

Royal 8vo., illustrated by a series of Original Photo-micrographs and many Illustrations in the Text, cloth gilt, **7s. 6d.** net,

### THE MENOPAUSE AND ITS DISORDERS.

**With Chapters on Menstruation.**

By A. D. LEITH NAPIER, M.D., M.R.C.P., late Editor of the *British Gynæcological Journal.*

"Of great practical use to the general practitioner, as well as to those more especially engaged in gynæcological work."—*The Hospital.*

London: THE SCIENTIFIC PRESS, LTD., 28 & 29, Southampton Street, Strand, W.C.

### Hospitals, &c., Urgently Needing Annual Subscriptions, Donations, and Legacies.

### LONDON HOSPITAL, E.

### The Committee appeal for £40,000 a-year from VOLUNTARY CONTRIBUTIONS.

The number of IN-PATIENTS treated in 1897 was 11,146
" OUT-PATIENTS " " 161,033
Total number of Patients treated at the Hospital—172,179

### FUNDS ARE URGENTLY NEEDED.

**Annual Subscribers are entitled to Tickets.**

Thoroughly-Trained Private Nurses to be had immediately on application to the Matron.

HONBLE. SYDNEY HOLLAND, *Chairman.* G. Q. ROBERTS, *House Governor.*

**THE UNIFORM SYSTEM OF ACCOUNTS, AUDIT, AND TENDERS, for Hospitals and Institutions, with Certain Checks upon Expenditure; also the Index of Classification.** Compiled by a Committee of Hospital Secretaries, and adopted by a General Meeting of the same, 18th January, 1892. And certain Tender and other Forms for securing Economy, By Sir HENRY BURDETT, K.C.B. Demy 8vo, profusely illustrated with Specimen Tables, Index of Classification, Forms of Tender, &c., cloth extra, 6s.

**ACCOUNT BOOKS FOR INSTITUTIONS, designed in accordance with the Uniform System of Accounts for Hospitals and Institutions.** By Sir HENRY BURDETT, K.C.B. Being a complete set of Account Books ruled in accordance with the Uniform System adopted by the Metropolitan Hospital Sunday Fund.

*(Full Description of Books and Prices sent post free on application.)*

London: THE SCIENTIFIC PRESS, Ltd., 28 & 29, Southampton Street, Strand, W.C.

### BOOKS ON NURSING.

**WOUNDED IN WAR.** Surgeon's Handbook of Treatment. By F. ESMARCH, translated by H. H. Chilton. With coloured plates and 536 engravings. 8vo., £1 8s.

**MANUAL OF FIRST AID.** Being a Text-book for Ambulance Classes and a Work of Reference for Domestic and General Use. By Dr. J. A. AUSTIN, Lecturer to St. John's Ambulance Association, and Author of "Ambulance Sermons." Illustrated with diagrams. Dedicated by permission to H.R.H. Princess Christian of Schleswig-Holstein. Crown 8vo., 3s. 6d. [*Ready.*

"Dr. Austin's little book is well and interestingly written. . . . The plan of the book follows the syllabus of the St. John's Ambulance Association, and is copiously illustrated with diagrammatic figures, which are plain and simple and accurate. Altogether the book is a good specimen of its class."—*Lancet.*

**A COMPLETE SYSTEM OF NURSING.** Written by various contributors, consisting entirely of Medical Men and Nurses. Edited by Honnor Morten, Author of "The Nurse's Dictionary," &c. 8vo., cloth, 7s. 6d.

"We are bound to say that the greater part of this handy volume contains much that is useful and interesting. . . . Lastly, the list of recipes for invalid cookery is both original and serviceable."—*The Nurses' Journal.*

"As a book of reference, a nurse may find in its pages exactly the information she may want to resolve a doubt or difficulty."—*Glasgow Herald.*

"The chapter on 'The Nursing of Babies' is admirable, and should be read by all mothers as well as nurses."—*The Hospital.*

**HOW TO TREAT ACCIDENTS AND ILLNESSES.** A Handbook for the Home. By HONNOR MORTEN, Author of "Sketches of Hospital Life," &c. Second Edition. With illustrations. Post 8vo., boards, 2s.; cloth, 2s. 6d.

"A very sensible 'handbook for the home,' prepared by a practical person, presumably a lady with nursing experience. It tells what to do in case of common accidents, common emergencies, and the various common illnesses to which flesh is heir. The explanations are given in the simplest business-like language, and the incidental advice is full of common-sense, and tends to reassurance of the patient. To the systematic treatment of the various physical troubles liable to afflict a family are appended some useful general hints on nursing and on the preservation of health."—*Daily Chronicle.* "Just such a book as a nurse or a good housewife needs."—*Scotsman.*

**LYING-IN.** A Book for Every Mother. By MARIAN HUMFREY, of the British Lying-In Hospital, London, and Author of "A Manual of Obstetric Nursing." Small crown 8vo., paper covers, 1s.

Dr. Alfred Hill (Medical Officer of Health, Birmingham) says:—"I have gone through the book, and am convinced that if lying-in women will follow its directions, the discomforts, dangers, and diseases which too often attend the natural process will be either avoided or mitigated."

**1. A MANUAL OF OBSTETRIC NURSING.** By MARIAN HUMFREY. Part I.—MATERNAL. Part II.—INFANTILE. Crown 8vo., cloth, 3s. 6d. Dedicated by permission to H.R.H. the late Princess Mary of Cambridge, Duchess of Teck.

*The Hospital* says: "This volume is certainly a very valuable addition to the literature of obstetric nursing. Not only is every little detail mentioned, but its special excellence consists in the very lucid manner in which all is explained. . . . The arrangement of the chapters, and the advice given are beyond praise."

**2. A MANUAL OF OBSTETRIC NURSING.** By MARIAN HUMFREY. Vol. II. Part I.—MATERNAL. Part II.—INFANTILE. Crown 8vo., cloth, 3s. 6d. This volume treats of duties, special to the gravest emergencies, of Obstetric Nursing, and thus completes the curriculum of instruction.

London: SAMPSON LOW, MARSTON & COMPANY, Limited., St. Dunstan's House, Fetter Lane, E.C.

**CLEANLINESS IN CHILDREN.**
By ROSE PETTY, Associate of the Sanitary Institute.
Crown 8vo. Paper cover, 2d.
London: The Scientific Press, Ltd., 28 & 29, Southampton Street Strand, W.C.

Accouchement Requisites.

Ambulance Cases.

### NOW READY,

A REPRINT OF

### A NURSING HANDBOOK

TOGETHER WITH S. MAW, SON, AND THOMPSON'S

### COMPLETE CATALOGUE OF NURSING REQUISITES.

The above work is now republished at a cost of 1s. per copy. Messrs. S. M., S., and T. have been compelled to make this charge on account of the **extreme** popularity of the work and the great expense incurred in its production.

***Post Free on receipt of 1s.***

### S. MAW, SON, and THOMPSON,

7 to 12, ALDERSGATE STREET, LONDON, E.C.

